# Quantifying Grain-Size Variability of Metal Pollutants in Road-Deposited Sediments Using the Coefficient of Variation

**DOI:** 10.3390/ijerph14080850

**Published:** 2017-07-28

**Authors:** Hongtao Zhao, Xiaoxue Wang, Xuyong Li

**Affiliations:** 1 State Key Laboratory of Urban and Regional Ecology, Research Center for Eco-Environmental Sciences, Chinese Academy of Sciences, Beijing 100085, China; htzhao@rcees.ac.cn (H.Z.); wangxiaoxue@ciecc.com.cn (X.W.); 2 China International Engineering Consulting Corporation, Beijing 100048, China

**Keywords:** road-deposited sediment, grain size variability, coefficient of variation, metal pollution

## Abstract

Particle grain size is an important indicator for the variability in physical characteristics and pollutants composition of road-deposited sediments (RDS). Quantitative assessment of the grain-size variability in RDS amount, metal concentration, metal load and *GSF_Load_* is essential to elimination of the uncertainty it causes in estimation of RDS emission load and formulation of control strategies. In this study, grain-size variability was explored and quantified using the coefficient of variation (*Cv*) of the particle size compositions, metal concentrations, metal loads, and *GSF_Load_* values in RDS. Several trends in grain-size variability of RDS were identified: (i) the medium class (105–450 µm) variability in terms of particle size composition, metal loads, and *GSF_Load_* values in RDS was smaller than the fine (<105 µm) and coarse (450–2000 µm) class; (ii) The grain-size variability in terms of metal concentrations increased as the particle size increased, while the metal concentrations decreased; (iii) When compared to the Lorenz coefficient (*Lc*), the *Cv* was similarly effective at describing the grain-size variability, whereas it is simpler to calculate because it did not require the data to be pre-processed. The results of this study will facilitate identification of the uncertainty in modelling RDS caused by grain-size class variability.

## 1. Introduction

Road surfaces have different mixes of sources contributing to the solids loadings in urban environments, and road-deposited sediment (RDS) is an important environmental medium [1,2,3]. The diverse sources of RDS make it a complex and heterogeneous mixture of organic and inorganic components [4,5]. RDS behaviour (e.g., mobilities and amounts of particles present, sources and sinks of RDS, and pollutant concentrations and species that are present, including the types of particles to which the pollutants are attached) depends on the sizes of the particles present [6,7,8]. For example, many contaminants, including metals, are found at higher concentrations in finer particles than in coarser particles [9], and finer particles are more likely to be resuspended in the air or entrained in rainfall runoff than coarser particles [7,10,11]. Overall, RDS behaviour in terms of the particle sizes present is attracting increasing attention [12,13].

It is well known that the particle grain size is a very important indicator because it links many aspects of the RDS. To avoid the unpredictability resulting from grain-size variability, it is essential to elimination of the uncertainty it causes in estimation of RDS emission load and formulation of control strategies [14,15]. Many studies have shown that RDS of a given size will vary in surface morphology, degree of aggregation, ratio of inorganic to organic content, and overall particle density [8,16,17,18]. Although there is a good deal of research on RDS that can be used when discussing grain-size class variability, no coefficient of variation has been found to quantify the inter-class variability in terms of important details regarding the actual RDS composition. A ‘black box’ approach is based system theory and often applied to research the cause and the effect. Generally, the cause is with very little knowledge and the approach maybe is suitable to investigate the appearance of grain-size variability due to little knowledge of particle itself hidden characteristics. The ‘black box’ approach applied in this study can be viewed in terms of the relations between the appearance of grain-size variability in RDS amount, metal concentration, metal load and *GSF_Load_* related to its other characteristics and behaviour within with very little knowledge of the particle sources, erosion and transport processes, mineral compositions, etc. Therefore, in this study, we used a ‘black-box’ approach to compare the inter-class variability, as measured by the coefficient of variation (*Cv*), in the amounts of particles present, particle sizes, and metal concentrations and loads in the particles. Moreover, the relationships between the variabilities in different particles size fractions and metal pollutants in RDS were identified.

## 2. Materials and Methods

### 2.1. Data Sources and Study Sites

Beijing lies between 39°26′ N and 41°03′ N and between 115°25′ E and 117°30′ E with an area of 16,808 km^2^. The region has 16 administrative sub-divisions, each of which is a county-level unit governed directly by the municipality. Six of the sub-divisions are urban districts, eight are suburban districts, and two are suburban counties. The Beijing region can be divided into urban, suburban, and rural areas, with counties containing suburban and rural areas each containing a group of towns, and each town consisting of a group of villages. We established five sampling areas along an urban–suburban–rural gradient, including sites in the central urban area (UCA), an urban village area (UVA), a central suburban county area (CSA), a rural town area (RTA), and a rural village area (RVA).

The metropolitan Beijing region is useful for studying variability in RDS and the metals associated with RDS on a regional scale because there are few point sources of heavy metals emissions in the region [19]. A total of 167 RDS samples were collected carefully along the urban–suburban–rural gradient, 97 from areas with busy main roads and 70 from residential areas. We accounted for the dependence of variations (i.e., population density, average daily traffic, street cleaning method, frequency of sweeping, etc.) in the RDS characteristics on the presence of roads in the sampling areas by conducting field investigations before selecting an appropriate number of sampling sites in each area. The numbers of sampling sites in each area along the urban-suburban-rural gradient are shown in Table 1.

RDS samples were collected using a domestic vacuum cleaner (Philips FC8264; Philips, Amsterdam, The Netherlands) between 2 and 10 September 2009, after the weather had been dry for about 2 weeks. This vacuum cleaner had high efficiency with an air filtration system and a cyclonic dustbin that effectively captures microscopic particulates. An unspecified area at each site was vacuumed from the central road marking to the curb until a reasonable amount of RDS had been collected, after which the size of the area sampled was measured using a ruler. Each RDS sample was weighed using an electronic balance, and the sample masses ranged from 0.8 to 1.5 kg. The mass of RDS per unit area was calculated by dividing the RDS mass collected by the size of the sampling area, and the values ranged from 2 to 570 g/m^2^ for all samples. In general, more RDS was generally collected from sites in the RTA, RVA, and UVA than from sites in the UCA and CSA. RDS particles were dry-sieved using polyester test sieves. Bulk particles were passed through square-holed sieves and fractionated into eight sub-samples <44, 44–62, 62–105, 105–149, 149–250, 250–450, 450–1000, and 1000–2000 μm. To maintain consistency of the sieving process, these parameters (time, amplitude of shaking, and interval) were maintained consistent to the greatest extent possible. We categorized the grain size classes of RDS into fine (<105 μm), medium (105–450 μm) and coarse (450–2000 μm) in this study. 

### 2.2. Analytical Methods and Quality Control

The total Cr, Cu, Ni, Pb, and Zn concentrations in each sample were measured after digesting the sample in a mixture of HF and HClO_4_ on a hotplate [20]. The quality assurance and quality control (QA/QC) procedures were conducted using the certified reference materials (CRMs), GSS-1 and GSS-2 (Geochemical Standard Soil) [21]. There were no CRMs available for RDS. The CRMs of soil used for quality control for RDS were acceptable for some published references [2,21]. Recoveries of the 5 observed metals were between 75–110% (75–95% for Cr, 90–108% for Cu, 90–104% for Ni, 90–110% for Pb, 98–107% for Zn). Detection limit of Cr, Cu, Ni and Zn ranged from 0.1 to 1 μg/L, and that of Pb ranged from 1 to 10 μg/L. Duplicates were analyzed on 2% of the RDS samples and the standard deviations were within ±10% of the mean. Reagent blanks were included with each batch of samples assayed.

### 2.3. Estimation of the Heavy Metal Loads in the RDS Samples

We determined the contributions of the metals in particles of different sizes to the overall metal concentrations in the RDS samples by calculating the load percentage for the metals in each RDS sample. The grain size fraction load (*GSF_Load_*) was calculated using Equation (1) [2]:

(1) $${\mathrm{GSF}}_{\mathrm{Load}}=\frac{C_{i}\times GS_{i}}{\sum_{i=1}^{n}C_{i}\times GS_{i}}\times 100$$

where, *C_i_* is the heavy metal concentration in grain size fraction *i* in a RDS sample (mg/kg), *GS_i_* is the percentage by mass that size fraction *i* contributed to the RDS sample (%), and *n* is the number of grain size fractions.

### 2.4. Quantification of Grain-Size Class Variability in RDS

The *Cv* is a dimensionless quantitative description of the degree of variability relative to the mean. Before calculating the *Cv*, the normal distribution of data in the present study was verified using SPSS 19.0 (IBM, Armonk, NC, USA). The most commonly used method for calculating the *Cv* is shown in Equation (2) [22]:

(2) $$Cv=\frac{\sqrt{\sigma^{2}}}{\ddot{x}}$$

where, $\sqrt{\sigma^{2}}$ is the standard deviation and $\ddot{x}$ is the mean. 

Statistical analyses were conducted using the R for Windows software package (R Core Team, 2012). The confidence intervals for the *Cv* values were determined using the Methods for Behavioral, Educational, and Social Sciences packagein the R for Windows software package [23]. The Gini index and Lorenz curve were determined using the tuxettechix (2012) (http://tuxette.nathalievilla.org/?p=508&lang=en) procedure in the R for Windows software package. 

## 3. Results

In this section, the *Cv* was used to quantify the variability or variability of particles of different sizes in terms of the amounts present, the metal concentrations and loads, and the *GSF_Load_* values. A larger *Cv* value indicates more variation in the parameter, which could reflect greater variability or heterogeneity [22]. The confidence interval of a *Cv* will be used to illustrate the expected accuracy with which the *Cv* parameter was estimated. We used RDS in areas near main roads (R1) and residential areas (R2) in Beijing that were determined in our previous studies [9,24]. All of the study sites were within the metropolitan Beijing area. 

### 3.1. Grain-Size Class Variability in Terms of the Amounts or Mass Percentage Present

**Figure 1:**
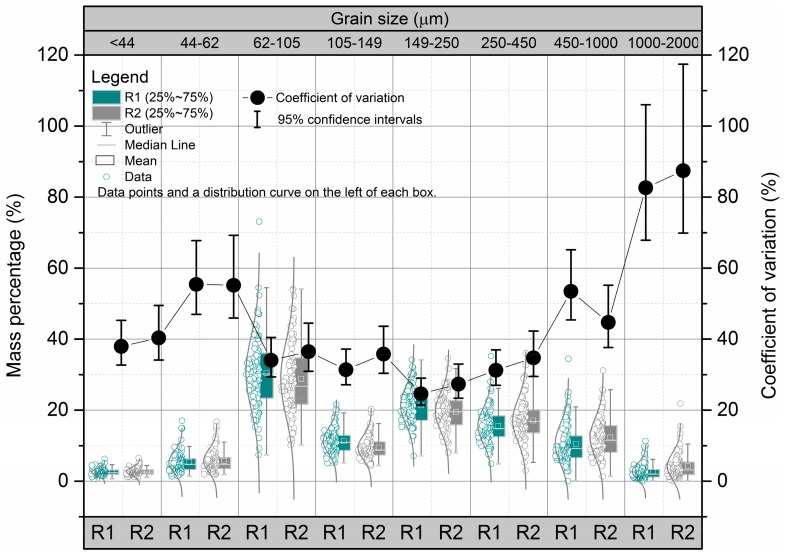
Grain-size heterogeneity in terms of the amounts present. Each *Cv* value shown is the mean ± the 95% confidence interval. R1 = areas in which there are main roads, R2 = residential areas.

The grain-size class variability in term of the contributions of individual particle sizes to the bulk particles (mass percentage, %) was measured using the *Cv*. As shown in Figure 1, the trends in the *Cv* values for the particles of different sizes were similar for the RDS samples from areas containing main roads (R1) and residential areas (R2). The individual particles mass percentage increased in the following order: <44 μm < 1000–2000 μm < 44–62 μm < 105–149 μm < 450–1000 μm < 250–450 μm < 149–250 μm < 62–105 μm. The grain-size class variability (*Cv* values) in terms of mass percentage increased in the following order: 149–250 μm < 105–149 μm ≈ 250–450 μm < 62–105 μm < <44 μm < 450–1000 μm < 44–62 μm < 1000–2000 μm. Overall, the medium class (105–149, 149–250, and 250–450 μm) variability in terms of the contributions of individual particle sizes to the bulk particles was smaller than the fine (<44, 44–62, and 62–105 µm) and coarse (450–1000 and >1000 μm) class. Additionally, RDS with coarser grain size had clearly lower metal concentrations and greater variability. This could be attributed to a number of factors, including variations in the origins of particulate matter, land use, road surface conditions, and street cleaning methods [5,9]. Finally, the *Cv* values were higher in R1 than in R2 for each particle size fraction except for the 44–62 μm and 450–1000 μm particles, for which there was no significant difference. 

### 3.2. Grain-Size Class Variability in Terms of the Metal Concentrations Present

**Figure 2:**
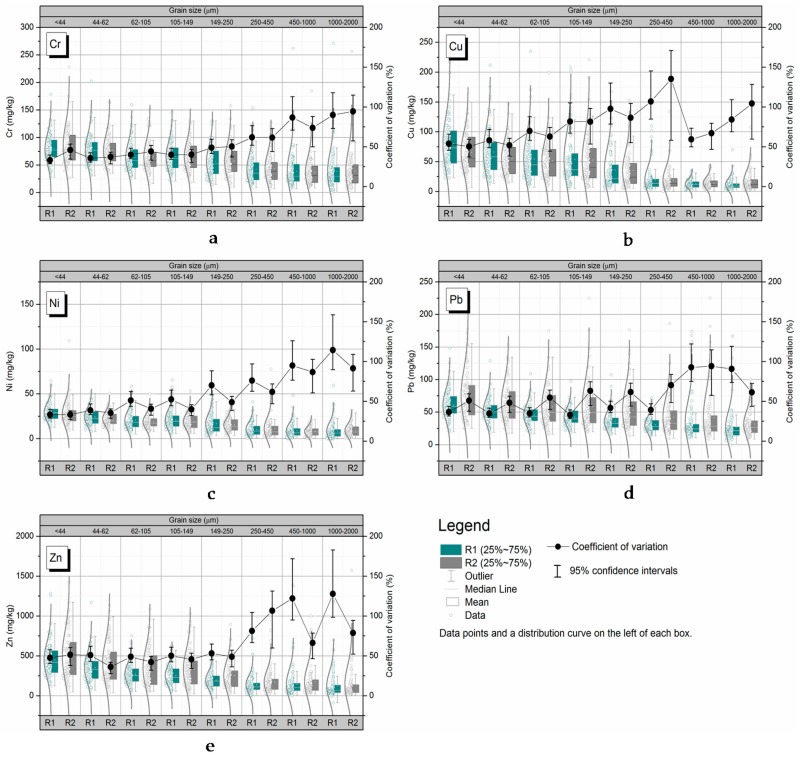
Grain-size heterogeneity in terms of metal concentrations present. Each *Cv* value shown is the mean ± the 95% confidence interval. R1 = areas in which there are main roads, R2 = residential areas; (**a**) grain-size variability in Cr concentrations; (**b**) grain-size variability in Cu concentrations; (**c**) grain-size variability in Ni concentrations; (**d**) grain-size variability in Pb concentrations; (**e**) grain-size variability in Zn concentrations.

Although the particle size distribution affected metal concentrations in RDS samples in our previous studies [9,24], few studies have investigated the variability of metal concentrations in individual grain size fractions. In the present study, the metal concentrations and their variabilities were found to be similar in the R1 and R2 samples (Figure 2). In general, the variabilities in metal concentrations increased as particle size increased because that RDS with coarser particles have great heterogeneity [13], whereas the metal concentrations decreased as particle size increased. The variabilities in the concentrations of all metals increased strongly as the particle size increased for particles with diameters >250 μm, with increases in Cu, Pb, and Zn variabilities being particularly sharply. The Cr concentration varied less with particle size than did the concentrations of the other metals. RDS samples with coarser grains were more variable than other RDS samples, which explained the high levels of variability in metal concentrations in the coarser samples relative to the metal concentrations in the same samples.

### 3.3. Grain-Size Class Variability in Terms of the Metal Loads Present

**Figure 3:**
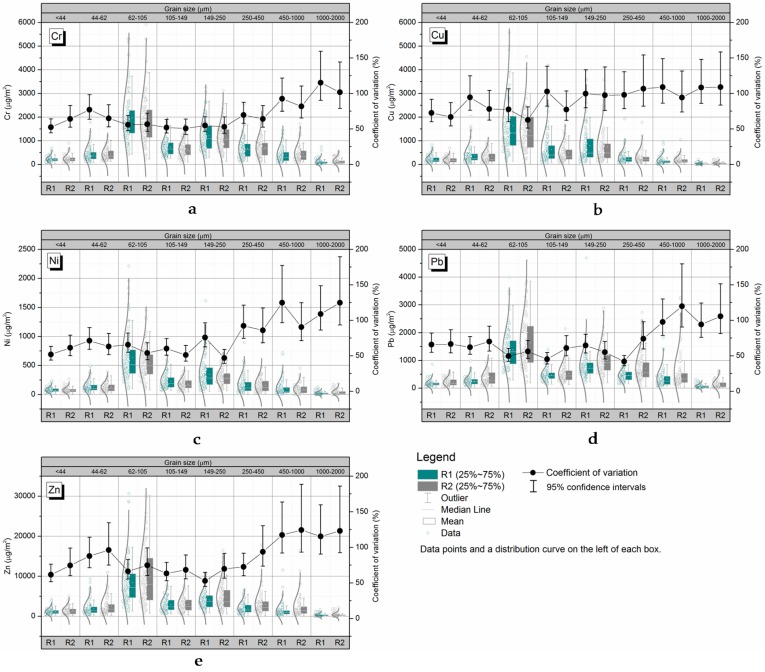
Grain-size heterogeneity in terms of metal load present. Each *Cv* value shown is the mean ± the 95% confidence interval. (**a**) grain-size variability in Cr loads; (**b**) grain-size variability in Cu loads; (**c**) grain-size variability in Ni loads; (**d**) grain-size variability in Pb loads; (**e**) grain-size variability in Zn loads.

Variability in particles of different sizes also had a strong effect on variations in metal loads. The metal loads in each grain size fraction in the R1 and R2 samples were more variable than the metal concentrations because the metal loads aggregate the variabilities in the amounts of particles present and the metal concentrations. As shown in Figure 3, the trends in the metal loads and the variabilities in the metal loads in R1 and R2 samples were similar. In general, the variabilities in the metal loads increased as the particle size increased, with the highest being found in particles with diameters of 62–105 μm. The loads of all metals except Cu increased greatly as particle size increased for particles with diameters of >250 μm. The Cu concentrations in the particles of different sizes varied less than the concentrations of the other metals. The variabilities in the metal loads in the particles of different sizes followed similar trends as the variabilities in the metal concentrations, even though the metal load in each size fraction is a combination of the amount of particles present and the metal concentration in those particles.

### 3.4. Grain-Size Class Variability in Terms of the GSF_Load_ Present

**Figure 4:**
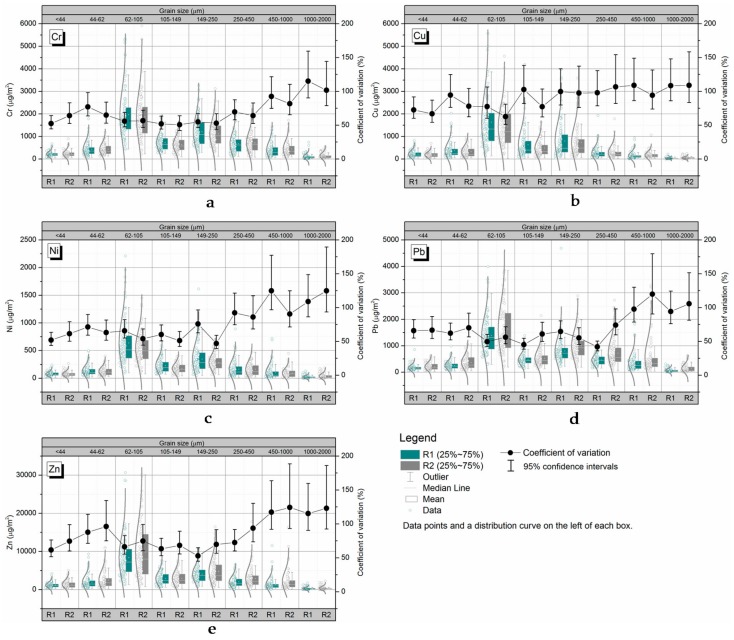
Grain-size heterogeneity in terms of *GSF_Load_* (%). *GSF_Load_* (%) is the contribution of the particles of a particular size to the overall level of metal contamination in a sample of sediment deposited on roads. Each *Cv* value shown is the mean ± the 95% confidence interval. (**a**) grain-size variability in Cr load; (**b**) grain-size variability in Cu *GSF_Load_* (%); (**c**) grain-size variability in Ni *GSF_Load_* (%); (**d**) grain-size variability in Pb *GSF_Load_* (%); (**e**) grain-size variability in Zn *GSF_Load_* (%).

The contributions of particles of different sizes to the overall levels of metal contamination in the R1 and R2 samples were estimated using the *GSF_Load_* values. As shown in Figure 4, the *Cv* values in the R1 and R2 samples followed similar trends. In general, relatively high *GSF_Load_ Cv* values were found for particles with diameters of >250 μm and <62 μm, while low *Cv* values were found for particles with diameters of 62–105 μm. The *GSF_Load_ Cv* values increased greatly as the particle size increased for particles with diameters of >250 μm, similar to the case for the metal concentration and load variabilities. Particles with diameters of 62–105 μm made the largest contributions to the overall level of metal contamination in the RDS samples, but had the least variable *GSF_Load_* values. In contrast, particles with diameters of 1000–2000 μm made the smallest contributions to the overall level of metal contamination in the RDS samples, but had the most variable *GSF_Load_* values.

## 4. Discussion

### 4.1. Impact of Grain-Size Class Variability on Quantification of RDS Washoff Loads

Obtaining accurate estimates of the parameters of RDS emission should be of the utmost concern when quantifying RDS washoff loads. The particle size distribution is an important parameter because it determines the mobility of the RDS and the pollutant concentration as particles of particular sizes are transported in runoff [6,25,26]. Therefore, grain-size heterogeneity or variability could strongly influence the accuracy with which particulate pollutant mass loss can be estimated [27,28]. Variability based on individual size classes should be explored and quantified to eliminate the inherent uncertainty it causes [29]. It is well known that many factors (e.g., road lay-out, road surface conditions, surrounding land use, traffic characteristics) determine the grain-size class variability on particles amount, pollutant species, pollutants concentration and chemical forms [30,31,32]. Most of the studies have shown that coarse particles had higher pollution levels (e.g., metals, PAHs, nutrients) than fine particles [2,21,33]. However, how to measure and quantify grain-size class heterogeneity is still unclear. In this study, we quantify the grain-size class variability measured by *Cv* from a ‘black box’ perspective. However, this approach masks some important details regarding the actual RDS composition. Therefore, further studies should be performed to better grasp the source apportionment, which will facilitate identification of appropriate management initiatives. Additionally, particular attention should be paid to variability in fine particles in RDS because they make large contributions to the RDS washoff load [7,21,34].

### 4.2. Comparison of Different Measures Used to Quantify Grain-Size Class Variability

A range of statistical techniques are available to characterise and quantify variability, but the appropriate statistical technique should be simple and easily applied to a dataset. The Lorenz coefficient (*Lc*) and the *Cv* were used in this study to help understand the effectiveness with which variabilities in particle sizes and RDS characteristics can be quantitatively estimated. The *Lc* and *Cv* values indicating the variabilities in the metal concentrations of particles of different sizes in the R1 samples are shown in Table 2. The *Lc* and *Cv* values were similarly effective at describing the variabilities. Both *Lc* and *Cv* were able to describe the variability of a dataset as a single value, allowing different data types to be compared directly. However, the *Cv* was found to have a major advantage over the *Lc* in that the *Cv* is simpler to calculate, does not require the data to be pre-processed, and can distinguish between extreme variations [23,35]. Nevertheless, the *Lc* can provide a simple graphical approach to visualizing and quantifying variability. Overall, the *Cv* could be used more easily than the *Lc* to quantitatively estimate the variabilities in particle sizes and RDS characteristics in this study. 

## 5. Conclusions

Based on a ‘black-box’ approach, the grain-size class variability in terms of mass percentage, metal concentration, metal load, and *GSF_Load_* were measured by the coefficient of variation (*Cv*). The results indicated that the medium class variability in terms of the contributions of individual particle sizes to the bulk particles was smaller than the fine and coarse class. The grain-size class variability in terms of metal concentration increased as the particle size increased, whereas the metal concentrations decreased as the particle size increased. The grain-size class variability in metal load aggregate the variabilities in the amounts of particles present and the metal concentrations, and it also increased as particle size increased. Particles with diameters of >250 μm and <62 μm have higher *GSF_Load_ Cv* values, and the *GSF_Load_ Cv* values increased greatly as the particle size increased for particles with diameters of >250 μm. The results of this study will improve our ability to evaluate and control the discharge of RDS in rainfall runoff by elimination of the uncertainty caused by grain-size variability in physical characteristics and pollutants composition of RDS.

## Figures and Tables

**Table 1:** Numbers of sampling sites in each area along the urban-suburban-rural gradient.

Study Site	Central Urban Area (UCA)	Central Suburban County Area (CSA)	Rural Town Area (RTA)	Rural Village Area (RVA)	Urban Village Area (UVA)
Areas containing main roads (R1)	11	40	20	20	6
Residential areas (R2)	8	18	20	20	4

**Table 2:** A comparison of two methods (the coefficient of variation, *Cv*, and the Lorenz coefficient, *Lc*) to quantify the grain-size class heterogeneity in metal concentration in RDS.

Particle Size (μm)	Cr		Cu		Ni		Pb		Zn	
	*Cv*	*Lc*	*Cv*	*Lc*	*Cv*	*Lc*	*Cv*	*Lc*	*Cv*	*Lc*
<44	33.3	0.183	53.8	0.286	33.3	0.178	36.5	0.197	47.6	0.252
44–62	35.6	0.190	58.2	0.310	38.8	0.212	34.8	0.188	50.9	0.271
62–105	40.1	0.218	69.8	0.356	51.1	0.267	35.0	0188	49.0	0.262
105–149	40.1	0.221	81.8	0.412	52.4	0.268	32.9	0.180	50.1	0.277
149–250	48.9	0.270	97.7	0.475	70.0	0.367	41.6	0.207	53.1	0.298
250–450	61.8	0.320	106.9	0.442	76.0	0.396	39.1	0.211	81.8	0.380
450–1000	86.8	0.375	59.3	0.320	95.0	0.465	92.8	0.370	122.0	0.468
1000–2000	90.0	0.401	84.32	0.386	114.4	0.514	90.9	0.359	127.8	0.557
